# A Journey on Extracellular Vesicles for Matrix Metalloproteinases: A Mechanistic Perspective

**DOI:** 10.3389/fcell.2022.886381

**Published:** 2022-05-20

**Authors:** Sylvie Thuault, Rania Ghossoub, Guido David, Pascale Zimmermann

**Affiliations:** ^1^ Centre de Recherche en Cancérologie de Marseille (CRCM), Equipe Labellisée Ligue 2018, CNRS, Inserm, Institut Paoli Calmettes, Aix-Marseille Université, Marseille, France; ^2^ Department of Human Genetics, KU Leuven, University of Leuven, Leuven, Belgium

**Keywords:** extracellular vesicles, exosomes, invadopodia, matrix metalloproteinases, trafficking

## Abstract

Matrix metalloproteinases (MMPs) are key players in matrix remodeling and their function has been particularly investigated in cancer biology. Indeed, through extracellular matrix (ECM) degradation and shedding of diverse cell surface macromolecules, they are implicated in different steps of tumor development, from local expansion by growth to tissue invasion and metastasis. Interestingly, MMPs are also components of extracellular vesicles (EVs). EVs are membrane-limited organelles that cells release in their extracellular environment. These “secreted” vesicles are now well accepted players in cell-to-cell communication. EVs have received a lot of interest in recent years as they are also envisioned as sources of biomarkers and as potentially outperforming vehicles for the delivery of therapeutics. Molecular machineries governing EV biogenesis, cargo loading and delivery to recipient cells are complex and still under intense investigation. In this review, we will summarize the state of the art of our knowledge about the molecular mechanisms implicated in MMP trafficking and secretion. We focus on MT1-MMP, a major effector of invasive cell behavior. We will also discuss how this knowledge is of interest for a better understanding of EV-loading of MMPs. Such knowledge might be of use to engineer novel strategies for cancer treatment. A better understanding of these mechanisms could also be used to design more efficient EV-based therapies.

## 1 Introduction

Extracellular matrix (ECM) remodeling plays a crucial role during development and later to maintain tissue homeostasis (Bonnans et al., 2014; Winkler et al., 2020). During cancer progression, tissue matrix is modified to create a microenvironment favoring tumorigenesis and metastasis, supporting tumor growth, migration and invasion, angiogenesis, and immune suppression. Tumor cells, in close collaboration with tumor-associated stromal cells, deposit an ECM that differs from that made by their normal counterparts, altering the biochemical composition of the surrounding microenvironment. By activating enzymes involved in crosslinking ECM components, they also modify the biophysical properties of the ECM. Increased ECM stiffness is correlated to tumor progression in multiple cancer types. Furthermore, tumor cells and stromal cells degrade ECM components, clearing environmental barriers and favoring mobility, but also releasing signaling molecules and activating cell surface receptors.

## 2 Matrix Metalloproteinases (MMPs)

Matrix metalloproteinases (MMPs) compose a large family of secreted and membrane-associated proteinases essential for ECM remodeling. In total, 23 members are present in humans. Six of them are membrane-associated MMPs (MT-MMP): MT1-, MT2-, MT3-, and MT5-MMP are transmembrane proteinases, whereas MT4- and MT6-MMP are GPI-anchored.

**FIGURE 1:**
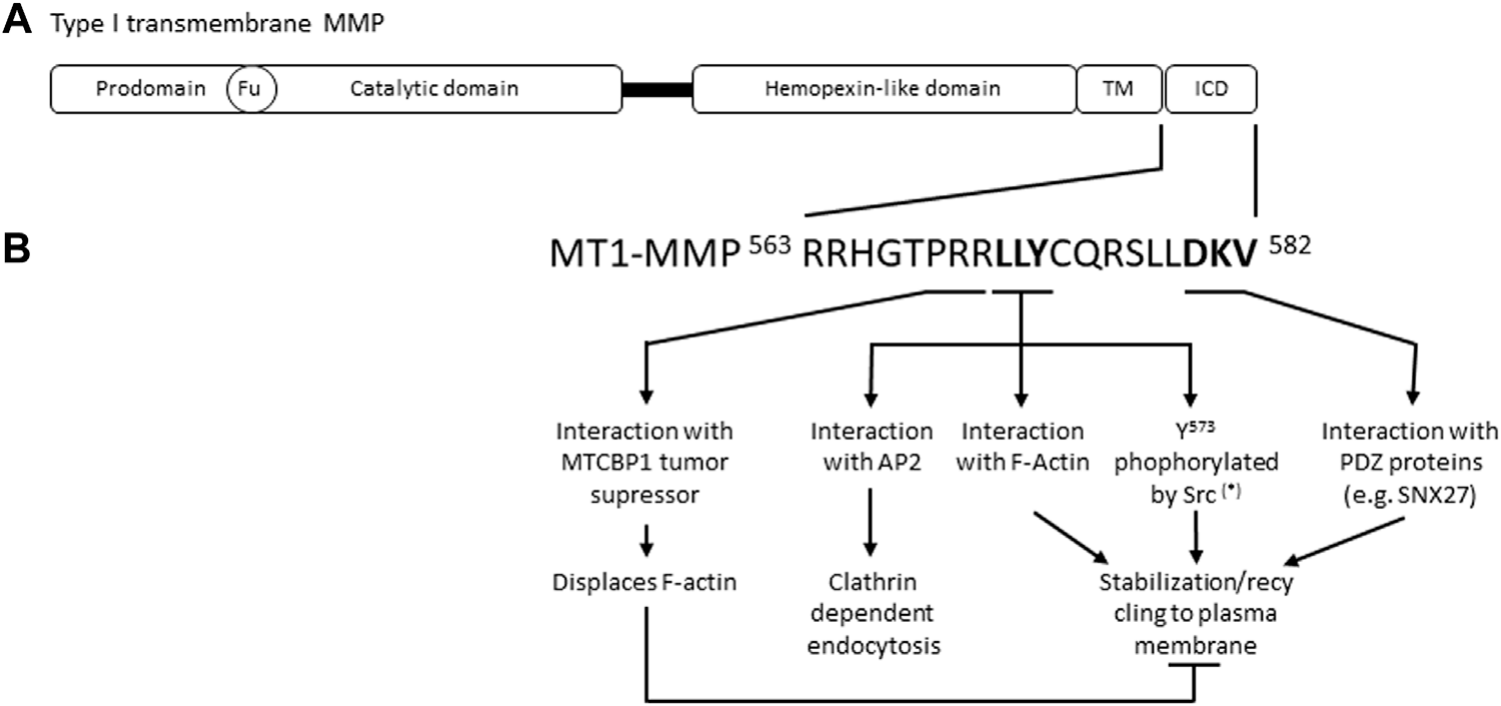
**(A)** Schematic representation of type I transmembrane MMPs. Fu, Furin cleavage recognition site; TM, transmembrane domain; ICD, intracellular domain. **(B)** Main MT1-MMP intracellular domain molecular features reported to control MT1-MMP endocytic and exocytic cycles. (*) other post-translational modifications affecting MT1-MMP stabilisation and recycling have been reported, please refer to the main text for further details. MTCBP1, MT1-MMP cytoplasmic tall-binding protein-1; AP-2, Adaptor Protein-2; F-Actin, filamentous actin; SNX27, Sorting Nexin 27.

MMPs share a common structure consisting of a pro-domain, a catalytic domain, and a C-terminal hemopexin-like domain (HPX) linked to the catalytic domain by a flexible serine rich region or linker peptide (Figure 1). Membrane-associated type I MMPs, such as MT1-MMP, contain a transmembrane domain and a short intracellular domain. MMPs are synthetized as inactive zymogens (pro-MMPs) and their activation requires a proteolytic cleavage that removes the pro-peptide. Indeed, this pro-domain contains a cysteine that interacts with the Zn^2+^ ion present in the catalytic domain, preventing enzymatic proteolytic activity (Van Wart and Birkedal-Hansen, 1990). Pro-domains are generally cleaved by other MMPs or serine proteases outside the cell, except for the transmembrane MMPs (MT-MMP), MMP-11 and MMP-28 which contain a furin recognition motif and are activated by intracellular furin-like serine proteinases. MMP activity can also be activated by oxidative stress, such as ROS, oxidizing the thiol cysteine group. In addition, MMP activity is regulated through 1) regulation of MMP expression, 2) trafficking and subcellular localization (internalization, recycling, secretion), 3) shedding, and 4) association with endogenous inhibitors (e.g., TIMPs, RECK). Regulatory steps depend on dimerization, post-translational modifications (e.g., phosphorylation, ubiquitination), and association with molecular partners. For further details on MMPs structure and activation see Brinckerhoff and Matrisian, 2002; Alaseem et al., 2019.

MMPs have some ECM substrate specificity. Together, MMPs degrade almost all the components of ECM (Bonnans et al., 2014). However, their activity is not limited to ECM components. For example, MT1-MMP is known to degrade ECM factors such as type I, II, III collagen, fibronectin, laminin-1 and -5, vitronectin, and aggrecan (Egeblad and Werb, 2002), but, in collaboration with the tissue inhibitor of matrix metalloproteinase family member TIMP-2, also cleaves the pro-peptide of pro-MMP2 and pro-MMP13, activating these enzymes. MT1-MMP also mediates the shedding of cell surface proteins such as CD44 (Kajita et al., 2001), *α*_*v*_ integrins (Deryugina et al., 2002; Ratnikov et al., 2002) and syndecans (Endo et al., 2003; Barbolina and Stack, 2008). MMPs can also cleave intracellular substrates, such as *α*-actinin-1 and 4, cofilin-1, filamins (Niland et al., 2021).

As key matrix endopeptidases, MMPs are implicated in diverse physiological processes such as embryogenesis, morphogenesis, and wound healing. Their deregulation is correlated with various pathological conditions, such as fibrotic diseases and cancer (Bonnans et al., 2014; Winkler et al., 2020). They are overexpressed in various types of cancer and are generally defined as bad prognostic factors, their expression increasing with cancer progression. Although generally pro-tumorigenic, some studies show anti-tumorigenic activities for some MMPs (Dufour and Overall, 2013). MMPs are expressed by cancer cells and tumor stromal cells, mainly cancer-associated fibroblasts, and endothelial cells. Their activities remodel the ECM, removing barriers and facilitating cell motility. They induce the production of short ECM fragments from long ECM molecules, called matrikines, acting as cytokines/chemokines. ECM degradation also allows the release and activation of matrix-bound growth factors. Thus, MMPs participate in the production of extracellular signaling molecules, modulating the activities of cell surface receptors, and thereby regulating signaling pathways implicated in cancer progression (Kessenbrock et al., 2010; Alaseem et al., 2019). MMPs have therefore been envisioned as therapeutical targets for cancer treatments. However, clinical trials are disappointing, due to the fact that inhibitors lack specificity, targeting both pro- and anti-tumorigenic MMPs (Dufour and Overall, 2013; Alaseem et al., 2019), reinforcing the need of a better understanding of the regulation of MMP activities.

## 3 Extracellular Vesicles

Intriguingly, MMPs were identified as extracellular vesicle (EV) cargoes (Shimoda and Khokha, 2017; Sanderson et al., 2019). EVs are membrane-limited organelles secreted by all types of cells in physiological and pathological conditions. EVs contain bioactive materials, such as proteins, nucleic acids and lipids, and enable the release of these materials in the extracellular environment through unconventional secretory pathways. Historically considered as “cell waste”, EVs are currently recognized as key actors in cell-to-cell communication (Tkach and Théry, 2016; Sato and Weaver, 2018; Kalluri and LeBleu, 2020). They act locally but also at a distance, circulating in almost all body fluids (e.g., blood, urine, saliva). EVs have received a lot of interest in recent years as they are envisioned as source of biomarkers, but also as promising vehicles for delivering therapeutics.

EVs are heterogeneous in terms of origin and size. Based on biogenesis, EVs can be classified in three major classes of EVs: apoptotic bodies, microvesicles and exosomes. Alternative nomenclatures refer to the method of purification. Apoptotic bodies are released upon cell death and will not be discussed in this review. Microvesicles (150 nm to a few µm), also called ectosomes or microparticles, emerge from outward budding of the plasma membrane. Exosomes (50–150 nm) have an endosomal origin. Intraluminal vesicles (ILV) are formed by an outward/away from the cytosol budding of the endosomal membrane during the maturation of multivesicular endosomes/bodies (MVB). Once ILVs are released in the extracellular microenvironment through fusion of MVBs with the plasma membrane, these are called exosomes. Diverse methods of fractionation allow the enrichment of the different EV subtypes and purification of specific EV subpopulations. For further details, please see Théry et al. (2018), Cocozza et al. (2020).

Molecular mechanisms supporting and regulating EV biogenesis, cargo loading and EV release are multiple and vary between cell types (Colombo et al., 2014; van Niel et al., 2018). These mechanisms, because not fully understood, represent a field of intensive research. The endosomal sorting complex required for transport (ESCRT) machinery is intimately implicated in ILV and MVB biogenesis. ESCRT-0 and ESCRT-I recruit cargoes at the limiting membrane of endosomes and then recruit successively ESCRT-II and ESCRT-III to allow the membrane budding and abscission that generate ILVs (Raiborg and Stenmark, 2009; Schmidt and Teis, 2012). The PDZ protein syntenin, due to its interaction with the accessory ESCRT protein ALIX and together with ESCRT components also regulates ILV biogenesis. The syntenin pathway is responsible for the loading of syndecan heparan sulfate (HS) proteoglycan and cargo bound to syndecan, e.g., FGFR, in exosomes (Baietti et al., 2012; Friand et al., 2015). Heparanase, an enzyme that cleaves HS chains internally, stimulates syntenin-syndecan-ALIX budding in ILVs leading to an increase in exosomal secretion (Roucourt et al., 2015). Interestingly, syntenin was recently proposed as universal exosome biomarker (Kugeratski et al., 2021). Lipids are also important regulators of ILV/exosome biogenesis and secretion (reviewed by (Egea-Jimenez and Zimmermann, 2020)). Indeed, several studies implicate ceramide, or its producing enzyme, neutral sphingomyelinase, in exosome secretion (Trajkovic et al., 2008). Phospholipase D2 and its product Phosphatidic Acid (PA), are also key players in exosome biogenesis and secretion (Ghossoub et al., 2014). Tetraspanins, more specifically CD9, CD63, and CD81, are common exosomal membrane components and can influence exosomal loading by clustering cargoes in specific membrane microdomains (van Niel et al., 2018). Yet tetraspanins can also inhibit exosome production, as illustrated for Tetraspanin-6 that reroutes MVB cargoes to lysosomal degradation (Ghossoub et al., 2020).

Different sub-populations of exosomes have been described to emerge from different endosomal compartments/trafficking routes (Colombo et al., 2014; Blanc and Vidal, 2018). Depending on the cell type, exosomes can emerge from Rab11/35 recycling endosomes, or Rab27 late endosomes. Molecular machineries implicated in MVB fusion with the plasma membrane have been identified. SNARE [Soluble *N*-ethylmaleimide-sensitive fusion attachment protein (SNAP) receptors] molecular machinery is widely implicated in vesicle fusion through formation of a complex between SNAREs present on the vesicles (v-SNAREs) and SNAREs present on the targeted membrane (t-SNAREs). The specific SNAREs involved in MVB fusion to plasma membrane, such as VAMP7 or SNAP23, vary depending on the cell type. Cortactin through its control of actin branching Arp2/3 complex activity and interaction with filamentous actin has also been involved in MVB fusion with plasma membrane (Sinha et al., 2016).

The biogenesis and release of microvesicles from the plasma membrane is influenced by phospholipid membrane constitution and actomyosin contractility (Clancy et al., 2021). In addition, some of the molecular machineries, including ESCRT machinery, used for MVB biogenesis have also been reported to be implicated in microvesicle budding and abscission from the plasma membrane (Hurley, 2015).

In the context of cancer, EVs are implicated in cancer cell growth, adhesion, motility, and invasion. They act on tumor cells but also on cells in the tumor microenvironment, promoting angiogenesis, dampening the immune system, and priming the metastatic niche (Becker et al., 2016; Peinado et al., 2017). Of importance, tumor cells have been shown to release significantly more EVs, compared to non-malignant cells, with numbers that increase with disease progression. Clearly, cancer EV cargoes are also different from normal cell EV cargoes. These alterations are triggered by diverse signals, coming from the tumor itself or from the tumor microenvironment, such as hypoxia or chemotherapeutic drugs (Bebelman et al., 2021). In breast cancer, the stiffness of ECM that is correlated to tumor progression has been directly implicated in the increase of EV secretion and cancer cell migration (Patwardhan et al., 2021). Finally, several *in vivo* studies indicate that depletion of EVs reduces tumor progression and metastasis (Peinado et al., 2012; Kosaka et al., 2013; Tickner et al., 2014; Costa-Silva et al., 2015; Nishida-Aoki et al., 2017). EVs have a direct impact on ECM. For example, cancer cells use EVs coated with the ECM component fibronectin as a substrate for directional migration (Sung et al., 2015; Purushothaman et al., 2016). EVs can also carry proteases either sticking at their surface or embedded in their membrane, and therefore have impact on ECM remodeling and cancer cell invasiveness.

## 4 MMPs in Extracellular Vesicles and Relation With Invadopodia

### 4.1 EV-Associated MMPs and Their Contribution in ECM Remodeling

MMPs have been identified, among other proteases, as associated with EVs of different tissue origins and in different physiological and pathological conditions (Taraboletti et al., 2002; Hakulinen et al., 2008; Muralidharan-Chari et al., 2009; Rossé et al., 2014). EV-associated MMPs control ECM remodeling and shedding of receptors located either at EV membranes or at the surface of targeted cells (Shimoda and Khokha, 2017; Sanderson et al., 2019; Shimoda, 2019). Furthermore, some MMPs, such as MMP3, have been described to be delivered *via* EV to recipient cell to act intracellularly (Okusha et al., 2020). Compared to the display of MT-MMPs at the cell surface and even the secretion of MMPs in the pericellular environment, EV-associated MMPs are suggested to be more performant at long distance ECM remodeling. EV-associated MMPs have thereby been implicated in activating stromal cells, angiogenesis, and pre-metastatic niche formation (Shimoda and Khokha, 2017). Intriguingly, the amount of EVs and of EV-associated MMPs correlates with the invasive potential of cancer cells (Ginestra et al., 1998; Di Vizio et al., 2012). These observations indicate that MMPs associated with EVs might be used as biomarkers of disease progression and responsiveness to anti-cancer treatments. Of interest, using a nanopatterned microchip, Zhang et al. were able to monitor tumor metastasis through analysis of EV-associated MT1-MMP levels (Zhang et al., 2020).

**FIGURE 2:**
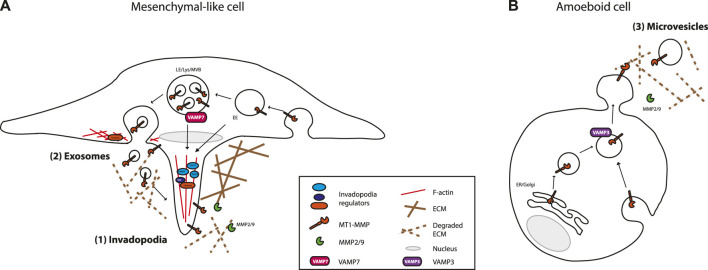
MT1-MMP main vesicular trafficking routes and sites of release/secretion. **(A)** Mesenchymal invading cells deliver MT1-MMP containing vesicles to degradative actin-rich membrane structures, called invadopodia (1), and in the extracellular microenvironment as associated to exosomes (2), after fusion of MVB with the invadopodial plasma membrane. Invadopodia are preferential sites of exosome secretion and exosomes potentiate invadopodia formation and proteolytic activity. **(B)** Amoeboid/blebbing invading cells release MT1-MMP associated to microvesicles (3) shed from the plasma membrane. Newly synthetised (not depicted for mesenchymal cells) and recycled MT1-MMP traffick to these different locations. The endosomal SNARE, VAMP-7 is implicated in the trafficking of MT1-MMP-containing vesicles to invadopodia and associated to exosomes, whereas the endosomal SNARE, VAMP-3 delivers MT1-MMP to microvesicles shed from the plasma membrane. Please refer to the main text for further details. LE, Late Endosome; Lys, Lysosome: MVB, Multi Vesicular Bodies; EE, Early Endosome.

Molecular machineries delivering MMPs in EVs are poorly understood. Yet, molecular mechanisms implicated in MMP delivery, especially that of MT1-MMP, to the extracellular space has been an intense field of research (Linder, 2007; Poincloux et al., 2009; Frittoli et al., 2011; Castro-Castro et al., 2016; Gifford and Itoh, 2019; Hey et al., 2021). MT1-MMP delivery to the extracellular microenvironment occurs through exocytosis at specialized plasma membrane domains such as lamellipodia and invadopodia, actin-rich cell protrusions with localized proteolytic activity generated by cancer cells (Figure 2). An intimate link between invadopodia and exosomes has been described. Below, we develop how these studies might provide a better understanding of MMPs loading in EVs and the biological impact of MMP present in EVs.

### 4.2 MMP Trafficking to Plasma Membrane and Invadopodia

MT1-MMP is considered as the major protease accounting for invadopodia proteolytic activity and has therefore been the major MMP studied. Studies of MT1-MMP trafficking to specific plasma membrane domains indicate that the recycling of MT1-MMP is important for MT1-MMP proteolytic activity and thereby its pro-invasive function (Linder, 2007; Poincloux et al., 2009; Frittoli et al., 2011; Castro-Castro et al., 2016; Gifford and Itoh, 2019; Hey et al., 2021).

#### 4.2.1 Importance of MT1-MMP Intracellular Domain in MT1-MMP Trafficking

MT1-MMP internalization, intracellular trafficking, plasma membrane recycling and degradation are mainly dictated by molecular determinants present in the short intracellular domain (20 amino acids) of MT1-MMP (Figure 1B). Some studies report that interaction of the extracellular hemopexin like domain of MT1-MMP with specific tetraspanins also regulates MT1-MMP trafficking and activity, positively and negatively, depending on the tetraspanin studied (Takino et al., 2003; Yañez-Mó et al., 2008; Lafleur et al., 2009; Schröder et al., 2013). Tetraspanin-enriched membrane domains act as platforms to selectively load specific cargoes in secretory MVBs (van Niel et al., 2018) and could be implicated in MT1-MMP loading in EVs.

MT1-MMP internalization is abrogated by MT1-MMP intracellular domain deletion (Nakahara et al., 1997; Lehti et al., 2000; Uekita et al., 2001). However, although MT1-MMP cell surface levels are increased and the enzyme is active, cells expressing this mutant MT1-MMP have impaired migratory and invasive capacities, indicating that MT1-MMP endocytosis/recycling/exocytosis cycles are important for MT1-MMP proteolytic activity (Remacle et al., 2003). More precisely, the LLY^573^ motif of MT1-MMP, interacting with the AP-2 clathrin adaptor, is required for MT1-MMP clathrin dependent endocytosis (Uekita et al., 2001). MT1-MMP is also internalized through other endocytic pathways, involving for example caveolae and flotillins, but the molecular features of MT1-MMP required for these types of endocytosis are not known (Remacle et al., 2003; Planchon et al., 2018). The metastasis-suppressor NME1 was recently reported to reduce the rate of MT1-MMP endocytosis in breast cancer cells by direct interaction with the cytoplasmic tail of MT1-MMP (Lodillinsky et al., 2021). Post-translational modifications of the MT1-MMP intracellular domain also influence its endocytosis. Phosphorylation of the Tyr^573^ by the kinases Src or LIMK has been reported to be required for MT1-MMP internalization (Nyalendo et al., 2007; Lagoutte et al., 2016). Phosphorylation of MT1-MMP Thr^567^ by protein kinase C (Moss et al., 2009; Williams and Coppolino, 2011) and palmitoylation of MT1-MMP Cys^574^ (Anilkumar et al., 2005) have also been described to promote MT1-MMP internalization and effects on cell invasion.

Interaction of MT1-MMP intracellular domain with filamentous actin (F-actin) is important for MT1-MMP endosomal trafficking and recycling. The LLY^573^ motif of the MT1-MMP C-terminal tail directly interacts with F-actin stabilizing MT1-MMP at degradative pseudopods of cells embedded in Matrigel (Yu et al., 2012). In contrast, interaction of the tumor suppressor MTCBP-1 (membrane-type 1 matrix metalloproteinase cytoplasmic tail-binding protein-1) with the PRR motif of MT1-MMP intracellular domain, displaces F-actin and inhibits invadopodia formation (Uekita et al., 2004; Qiang et al., 2019). MT1-MMP interaction with endosomal F-actin was also suggested to counteract MT1-MMP lysosomal degradation following the recruitment of the ESCRT-0 subunit Hrs to endosomal MT1-MMP-containing vesicles (MacDonald et al., 2018). The molecular mechanisms controlling the lysosomal degradation of MT1-MMP versus its recycling to plasma membrane/invadopodia deserve further studies.

The extreme C-terminal part of MT1-MMP intracellular domain corresponding to a class III PDZ binding motif (DKV^582^) plays a major role in MT1-MMP recycling (Figure 1B). Pioneer studies indicated that the PDZ binding motif of MT1-MMP was required for MT1-MMP recycling without affecting its internalization (Wang X. et al., 2004). More recently, the PDZ protein Sorting Nexin 27 (SNX27) was reported to interact with MT1-MMP PDZ binding motif allowing the recruitment of the retromer complex to MT1-MMP containing Rab7a-positive endosomes and enabling MT1-MMP recycling to invadopodia (Sharma et al., 2020). Intriguingly, SNX27 does not interact with MT2-MMP although MT2-MMP also contains a class III PDZ binding motif (EWV) (Pei, 1999; Sharma et al., 2020). The PDZ domain containing LIMK kinase also interacts with MT1-MMP PDZ binding motif, this interaction being required for MT1-MMP Tyr^573^ phosphorylation and cortactin accumulation to MT1-MMP endosomal vesicles (Lagoutte et al., 2016). Multiple different PDZ domain containing proteins interact with MT-MMP PDZ binding motifs regulating their activity and trafficking (Wang P. et al., 2004; Roghi et al., 2010). These results suggest that PDZ protein networks could be envisioned as fine tuners of MT-MMPs trafficking. Furthermore, monoubiquitination of MT1-MMP at Lys^581^ was found to depend on Src activity and to be necessary for MT1-MMP recycling to the plasma membrane (Eisenach et al., 2012).

Overall, these studies indicate that the LLY^573^ motif of the MT1-MMP C-terminal tail plays a major role in the regulation of MT1-MMP endocytosis and MT1-MMP stabilization at plasma membrane actin-rich domains, whereas the extreme C-terminal PDZ binding motif (DKV^582^) of MT1-MMP is mainly involved in its recycling.

#### 4.2.2 Molecular Machineries Implicated in MT1-MMP Endosomal Trafficking

Not surprisingly, Rab GTPases, key players in endosomal trafficking, play crucial roles in MT1-MMP delivery to specialized plasma membrane domains. The late endosome/lysosome (LE/Lys) Rab7- and LAMP1-positive endosomal compartment appears to be acting as a major MT1-MMP reservoir, albeit MT1-MMP recycling to the plasma membrane also occurs from early endosomes. Delivery of these MT1-MMP containing vesicles to invadopodia is dependent on the exocyst complex (Sakurai-Yageta, 2008; Monteiro, 2013), the retromer (Sharma et al., 2020), as well as different SNAREs. VAMP7 (Ti-VAMP) v-SNARE present on LE/Lys vesicles containing MT1-MMP, in concert with SNAP23 and Syntaxin4, is required for MT1-MMP delivery to invadopodia (Miyata et al., 2004; Steffen et al., 2008; Williams et al., 2014). SNAP23/Syntaxin13/VAMP3 are also involved in MT1-MMP trafficking to the plasma membrane (Kean et al., 2009). In LOX melanoma cells, however, VAMP3 is not required for MT1-MMP delivery to invadopodia, but is for MT1-MMP delivery to microvesicles, i.e., EVs directly shed from the plasma membrane (Clancy et al., 2015). VAMP3-specific loading of MT1-MMP into microvesicles is suggested to depend on the interaction of MT1-MMP with CD9, a tetraspanin implicated in the sorting of specific EV cargoes (Clancy et al., 2015). For more details on the regulation of MT1-MMP trafficking, we refer the readers to seminal reviews on the subject (Poincloux et al., 2009; Frittoli et al., 2011; Castro-Castro et al., 2016; Gifford and Itoh, 2019; Hey et al., 2021).

Why MT1-MMP recycling is important for regulation of MT1-MMP proteolytic activity is not fully understood. Fluorescent recovery after photobleaching (FRAP) experiments indicate that MT1-MMP associated with invadopodia is less mobile than MT1-MMP located in non-invadopodial regions of the plasma membrane (Yu et al., 2012). Thus, polarized recycling of MT1-MMP to invadopodial actin-rich plasma membrane domains would somehow permit MT1-MMP stabilization. We can also surmise that MT1-MMP recycling ultimately also favors MT1-MMP release as an exosome-associated factor.

### 4.3 Functional Interplay Between Invadopodia and Exosomes

Invadopodia are dynamic degradative actin-rich membrane protrusions elaborated by various cancer cells (Linder et al., 2011; Murphy and Courtneidge, 2011; Eddy et al., 2017). Their physiological counterparts, called podosomes, are elaborated by specialized normal cells, such as macrophages, monocytes, endothelial cells, and osteoclasts. Invadopodia and podosomes allow pericellular ECM proteolysis. In the context of cancer, invadopodia are required for tumor cells to break the basement membrane and to invade through interstitial matrix. They are therefore seen as key players in cancer cell invasiveness and metastasis. Although podosome and invadopodia morphologies differ, they share a common machinery necessary for their degradative function. Indeed, these structures are composed of structural and signaling proteins such as cortactin, cofilin, N-WASP, Arp2/3, Tks4/5 that control the reorganization of the actin cytoskeleton, and the release of proteases involved in matrix degradation (Linder et al., 2011; Murphy and Courtneidge, 2011). Invadopodia formation is a multistep process: 1. initiation, 2. assembly, 3. maturation and 4. disassembly. Firstly, diverse signals, such as growth factors and ECM stiffness, induce actin cytoskeleton reorganization leading to the formation of precursor invadopodia devoid of degradative activity. Then the precursor invadopodia are stabilized and serve as platforms for the recruitment of MMP-containing vesicles, leading to a mature, fully functional invadopodium.

Molecular machineries implicated in secretory MVB fusion with the plasma membrane and in delivery of MT1-MMP-containing vesicles to invadopodia are overlapping. For example, the SNAREs Ti-VAMP/VAMP7 and SNAP23 are necessary for delivery of MT1-MMP-containing vesicles to invadopodia (Steffen et al., 2008; Williams et al., 2014) and are also implicated in secretory MVB fusion with the plasma membrane (Fader et al., 2009; Wei et al., 2017) (Figure 2). Another example is cortactin. Cortactin, through its function as an activator of the branched actin nucleator Arp2/3 complex and binder of F-actin, is necessary for the formation of invadopodial membrane protrusions (Artym et al., 2006). In some cell types, cortactin is also required for the recruitment of MT1-MMP containing vesicles to invadopodia to permit their maturation (Clark et al., 2007). Further studies have shown that cortactin is more generally implicated in vesicular trafficking, localizing at the surface of endosomes and at the cell cortex (Kirkbride et al., 2011). In collaboration with Rab27a, cortactin was shown to participate in MVB docking to invadopodia (Sinha et al., 2016).

Furthermore, Hoshino et al. demonstrated that invadopodia and exosomes are intimately linked (Hoshino et al., 2013). Indeed, invadopodia were identified as preferential docking sites for CD63- and Rab27a-positive MVBs. Also, mechanistically, invadopodia formation and exosome secretion are somehow related. Indeed, inhibition of invadopodia formation, by means of Tks5, N-WASP or cortactin depletion, inhibited exosome secretion (Seals et al., 2005; Murphy and Courtneidge, 2011; Hoshino et al., 2013; Sinha et al., 2016). Inversely, invadopodia induction, through expression of a constitutively active form of PI3K (Yamaguchi et al., 2011), enhanced exosome secretion (Hoshino et al., 2013). Impact of the machinery implicated in invadopodia formation on MVB biogenesis was not directly addressed, so we cannot conclude whether the observed effects are reflecting impact on MVB formation or on MVB fusion with invadopodia-specific plasma membrane domains. This also raises the question of what plasma membrane domains compose preferential docking sites for MVBs, if any exist, in cells not forming invadopodia. Could these be secreted at lamellipodia which are also actin-rich structures? Reciprocally, inhibition of exosome production, through Hrs/ESCRT depletion or sphingomyelinase inhibition, the two main pathways implicated in MVB biogenesis, or inhibition of vesicle secretion, through depletion of Rab27a or Synaptotagmin-7, two factors implicated in MVB docking to plasma membrane, inhibited invadopodia formation (Hoshino et al., 2013). Overall, invadopodia seem to be required for exosome secretion, and exosome secretion be required for invadopodia formation in cells forming invadopodia (or to go hand in hand). However, induction of exosome secretion, through overexpression of Rab27b (Ostrowski et al., 2010), does not seem to be sufficient to induce invadopodia formation in cells that do not form invadopodia, such as MCF7 cells (Beghein et al., 2018). Interestingly, exosome-enriched fractions were able to potentiate invadopodia formation and stability (Hoshino et al., 2013). Intriguingly, ECM stiffness has been shown to increase invadopodia formation (Alexander et al., 2008) and to enhance exosome secretion and modify exosome contents (e.g., MMPs) (Patwardhan et al., 2021), supporting the notion of an intricate relationship between invadopodia and exosomes.

Melanoma cancer cells form invadopodia when seeded on rigid matrix (adopting a mesenchymal-like phenotype) and release less microvesicles (i.e., EVs-enriched in 10 000g pellets) than the same cells adopting an amoeboid-like phenotype when seeded on more compliant matrix (Sedgwick et al., 2015). Exosome release by amoeboid-like cells, however, has not been carefully analyzed. It is worth noticing, that even though amoeboid type of migration seems to be less dependent on ECM proteolysis compared to mesenchymal type of migration (Wolf et al., 2003; Wolf and Friedl, 2011; Orgaz et al., 2014), the degradative potential of microvesicles shed by amoeboid-like cells is high (Muralidharan-Chari et al., 2009; Sedgwick et al., 2015). This suggests that MMPs present on microvesicles might influence the invasive potential of tumor microenvironment cells rather than of the tumor cells themselves.

## 5 Concluding Remarks

MMPs are important for ECM remodeling during physiological processes and in pathological conditions, such as cancer. MMPs are exposed to the microenvironment at two main locations: 1) the cell surface, at specialized plasma membrane domains, such as invadopodia of cancer cells, and 2) associated to EVs (Figure 2). We can envision that cell surface-associated MMPs and EV-associated MMPs have distinct activities. MMPs associated with cell surfaces, through pericellular ECM remodeling, might obviously have a major autocrine function, whereas EV-associated MMPs might act mainly at distance influencing tumor microenvironment cells activities rather than influencing the producing cell activities. To address this point, it would be necessary to be able to follow EV-associated MMP activity *in vivo*. Furthermore, ECM composition and biophysical properties seem to influence the subtype of EVs released by a cell (i.e., exosomes versus microvesicles) (Figure 2). Cells evolving in a compliant matrix would preferentially release microvesicles, whereas cells evolving on stiffer matrix would release exosomes through invadopodia. Thus, cancer cells release MMPs in EVs, in addition to, but independently from, the secretion of MMPs involved in local tissue invasion. This suggests that a prominent role of EV-associated MMPs could be to influence tumor microenvironment at a distance, and, taking advantage of their circulation in body fluids, priming of the pre-metastatic niche. An intimate relationship exists between exosome secretion and invadopodia. This suggests that molecular features of the MT1-MMP intracellular domain implicated in MT1-MMP internalization and trafficking in different endosomal compartments before its release at invadopodia, might also be implicated in MT1-MMP loading in ILVs of MVBs. It would thereby be of interest to analyze the contribution of factors implicated in the loading of specific cargoes in EVs, such as the syntenin/ALIX pathway, and tetraspanin-enriched microdomains, in the loading of MT1-MMP/MMPs in EVs. This knowledge could be used to design molecules that would restrain MT1-MMP presentation at the cell surface or at the surface of EVs with the aim to inhibit the pro-tumorigenic activity of EVs. We could also use such knowledge to engineer EVs with an enhanced capacity to degrade the ECM and thereby EVs with a higher capacity to deliver therapeutics embedded in EVs.
